# Liver protective effect of ursodeoxycholic acid includes regulation of ADAM17 activity

**DOI:** 10.1186/1471-230X-13-155

**Published:** 2013-10-30

**Authors:** Halka Buryova, Karel Chalupsky, Olga Zbodakova, Ivan Kanchev, Marketa Jirouskova, Martin Gregor, Radislav Sedlacek

**Affiliations:** 1 Laboratory of Transgenic Models of Diseases, Institute of Molecular Genetics of the ASCR, v. v. i., Videnska 1083, Prague CZ142 20, Czech Republic

**Keywords:** Ursodeoxycholic acid, ADAM17, Shedding, Cholestasis, Liver

## Abstract

**Background:**

Ursodeoxycholic acid (UDCA) is used to treat primary biliary cirrhosis, intrahepatic cholestasis, and other cholestatic conditions. Although much has been learned about the molecular basis of the disease pathophysiology, our understanding of the effects of UDCA remains unclear. Possibly underlying its cytoprotective, anti-apoptotic, anti-oxidative effects, UDCA was reported to regulate the expression of TNFα and other inflammatory cytokines. However, it is not known if this effect involves also modulation of ADAM family of metalloproteinases, which are responsible for release of ectodomains of inflammatory cytokines from the cell surface. We hypothesized that UDCA modulates ADAM17 activity, resulting in amelioration of cholestasis in a murine model of bile duct ligation (BDL).

**Methods:**

The effect of UDCA on ADAM17 activity was studied using the human liver hepatocellular carcinoma cell line HepG2. Untransfected cells or cells ectopically expressing human ADAM17 were cultured with or without UDCA and further activated using phorbol-12-myristate-13-acetate (PMA). The expression and release of ADAM17 substrates, TNFα, TGFα, and c-Met receptor (or its soluble form, sMet) were evaluated using ELISA and quantitative real-time (qRT) PCR. Immunoblotting analyses were conducted to evaluate expression and activation of ADAM17 as well as the level of ERK1/2 phosphorylation after UDCA treatment. The regulation of tissue inhibitor of metalloproteinases-1 (TIMP-1) by UDCA was studied using zymography and qRT-PCR. A mouse model of acute cholestasis was induced by common BDL technique, during which mice received daily orogastric gavage with either UDCA or vehicle only. Liver injury was quantified using alkaline phosphatase (ALP), relative liver weight, and confirmed by histological analysis. ADAM17 substrates in sera were assessed using a bead multiplex assay.

**Results:**

UDCA decreases amount of shed TNFα, TGFα, and sMet in cell culture media and the phosphorylation of ERK1/2. These effects are mediated by the reduction of ADAM17 activity in PMA stimulated cells although the expression ADAM17 is not affected. UDCA reduced the level of the mature form of ADAM17. Moreover, UDCA regulates the expression of TIMP-1 and gelatinases activity in PMA stimulated cells. A BDL-induced acute cholangitis model was characterized by increased relative liver weight, serum levels of ALP, sMet, and loss of intracellular glycogen. UDCA administration significantly decreased ALP and sMet levels, and reduced relative liver weight. Furthermore, hepatocytes of UDCA-treated animals retained their metabolic activity as evidenced by the amount of glycogen storage.

**Conclusions:**

The beneficial effect of UDCA appears to be mediated in part by the inhibition of ADAM17 activation and, thus, the release of TNFα, a strong pro-inflammatory factor. The release of other ADAM17 substrates, TGFα and sMet, are also regulated this way, pointing to a general impact on the release of ADAM17 substrates, which are pivotal for liver regeneration and function. In parallel, UDCA upregulates TIMP-1 that in turn inhibits matrix metalloproteinases, which destroy the hepatic ECM in diseased liver. This control of extracellular matrix turnover represents an additional beneficial path of UDCA treatment.

## Background

Ursodeoxycholic acid (UDCA, 3α,7β-dihydroxy-5 β-cholanic acid) is an approved drug for the treatment of primary biliary cirrhosis and is also used to treat several other cholestatic conditions. It has also been reported to have beneficial effects for liver transplantation and some diseases not related to liver (for review see [1]). All data obtained so far suggest at least four mechanisms of action of UDCA in cholangiopathies: 1) an increased solubility of endogenous bile acids; 2) stimulation of hepatocellular and ductular secretions; 3) cellular protection against bile acid- and cytokine- induced injury; and 4) anti-inflammatory effects.

Specific effects of UDCA involve regulating the expression of basolateral bile salt transporters Mrp3 and Mrp4 [2] and the activity of the Cl^-^/HCO_3_ anion exchanger AE2 [3]. UDCA also protects cells against apoptosis [4] and counteracts the mitochondrial permeability transition induced by hydrophobic bile acids [5], and thus also the activation of caspases [6], death receptors, and apoptosis induced by endoplasmic reticulum stress [7]. At the molecular level, these multiple mechanisms of UDCA action include direct scavenging of reactive oxygen species (ROS) [8], increased transcription of antioxidant defense genes [9], stabilization of the plasma membrane against cytolysis [10] and reduction of p53 half-life by promotion of its ubiquitination and proteasomal degradation [11]. Another proposed mechanism implies beneficial anti-inflammatory effects, as UDCA treatment prevents hepatocytes from necrosis [12], thus reducing the local inflammatory response. This observation was confirmed in rats with bile duct ligation where liver injury is associated with leucocyte-dependent inflammation mediated by the release of pro-inflammatory cytokines [13].

The activity of metalloproteinases of the ADAM family is responsible for release (known as shedding) of membrane-associated cytokines, growth factors and their receptors, and adhesion molecules. This shedding process determines bio(un)availability of the factors and associated signaling during liver injury [14]. For instance, ADAM17 is responsible for shedding of several dozen cell-surface molecules, including the ligands of the epidermal growth factor receptor (EGFR), heparin-binding epidermal growth factor (HB-EGF), TNFα and its receptors (TNFR) (for review see [15]). Previous studies have shown that pharmacologic inhibition of ADAM17 abrogates inflammatory responses and has therapeutic potential in a variety of pathological conditions [16,17]. Interestingly, administration of marimastat, a broad spectrum inhibitor of matrix metalloproteinases (MMPs) and ADAM17, resulted in decreased fibrogenesis during repeated hepatotoxin-induced liver injury, acting presumably via the TNF-signaling pathway [18]. Analysis of mice deficient for TIMP-3, the endogenous inhibitor of ADAM17 [19], revealed elevated levels of TNFα and development of severe inflammation of the liver, presumably due to an increase in TNFα converting enzyme activity, i.e. the activity of ADAM17 [20].

Based on the fact that ADAM17 is a master regulator of bioavailability of cell-surface bound factors such as TNFα and TGFα, and the UDCA treatment modulates the levels of TNFα and other proinflammatory factors [21,22], we investigated whether UDCA-dependent alteration of TNFα, TGFα, and sMet levels is controlled via affecting ADAM17 proteolytic activity.

## Methods

### cDNA constructs and cloning

The human cDNA clone of full-length ADAM17 in the pCMV6-XL4 vector was obtained from OriGene (SC316426; OriGene Technologies, Rockville, MD). For the ectopic expression of untagged versions of ADAM17, cDNA was PCR-amplified from original plasmids and subcloned into the multiple cloning site 1 (MSC1) of the pVitro2-blasti plasmid (InvivoGen, San Diego, CA). The TdTomato and EGFP coding sequences were then amplified from plasmids (pEGFP; Clontech, Palo Alto, CA and pRSET-B-tdTomato; kindly provided by Roger Tsien, UC San Diego, USA) and subcloned into MSC2 of either pVitro-ADAM17 or pVitro-ADAM10 vector to generate pVitro-ADAM17-TdTomato construct encoding ADAM17 and reporter proteins under the control of a composite ferritin promoter. All plasmids were verified by sequencing.

### Cell culture experiments

The immortalized human liver hepatocellular carcinoma cell line HepG2 and the human hepatic stellate cell line LX2 (a kind gift of Scott Friedman, Mount Sinai, NY) were grown in Dulbecco's Modified Eagle's Medium (DMEM; Sigma Aldrich, St. Louis, USA) medium, supplemented with 10% heat-inactivated fetal bovine serum and 1% penicillin/streptomycin (both PAA Laboratories, Colbe, Germany). Cells were cultured at 37°C in 5% CO_2_ and routinely passaged every third day. To obtain subconfluent cultures (~80% confluence) for further experiments, cells were seeded at 20 × 10^3^ cells/cm^2^ in 6-well culture plates (Costar, Cambridge, MA). Cells were either left untreated or pretreated with 200 μmol/l UDCA (Sigma Aldrich, St. Louis, USA) alone or with 10 nmol/l metalloproteinase inhibitor TAPI-2 (EMD-Millipore, Billerica, USA) for 2 hours. Cells were then stimulated with 10 nM PMA (Sigma- Aldrich, St. Louis, USA) for an additional 24 hours.

Transient transfections of HepG2 and LX2 cells were carried out in serum free media using X-tremeGENE HP (Roche, Mannheim, Germany) according to the manufacturer’s instructions with 2 μg plasmid and a 1:3 (w/v) ratio of DNA to transfection reagent. Cells were incubated with the transfection complexes for 48 hours and assayed as above after an additional 24 h in media supplemented with 10% heat-inactivated fetal bovine serum and 1% penicillin/streptomycin. Conditioned media were collected and centrifuged at 12 000 × g for 15 minutes at 4°C. Supernatants were analyzed for TGFα and TNFα using colorimetric ELISA assays (R&D, Minneapolis, USA) and an EnVision Multilabel Reader (PerkinElmer, Waltham, USA). Quantitative cell fractionation of non-treated and UDCA-treated HepG2 cells was performed as before [23].

### Quantitative reverse-transcriptase polymerase chain reaction (qRT-PCR)

Total RNA was isolated from snap-frozen liver samples or cell cultures using TriReagent (Sigma-Aldrich, St. Louis, USA) according to the manufacturer’s instructions. RNA concentration was determined using a Nanodrop ND-1000 (Thermo Scientific Wilmington, USA). Unique primers were designed for ~100 bp segments of target gene transcripts using QuantPrime online software; Table 1). qRT-PCR was carried out directly from isolated RNA using Kapa SYBR Fast One-Step qRT-PCR Kit (Kapa Biosystems, Boston, USA) on a LightCycler 480 (Roche, Mannheim, Germany). Triplicate reactions were performed with the following conditions: 95°C for 3 min, followed by 40 cycles of 95°C for 30 sec, 60°C for 30 sec, and 72°C for 30 sec. The standard curve method was used to determine relative mRNA abundance. The relative mRNA levels were calculated by comparative Ct method as before [24] using glyceraldehyde-3-phosphate dehydrogenase (GAPDH) as the control and expressed as fold change of control sample (arbitrarily set to 1).

**Table 1:** Primer sequences for RT-PCR

	**Forward (5′-3′)**	**Reverse (5′-3′)**
c-Met	ACTTGGCTGCAAGAAACTGTAC	TTCACTGGCAGCTTTGCACCTG
CA	AGCACTGCCAGCAACAAGTCAG	ACGGATTGAAGGAGCCCATCTC
TNFα	CCAGGCAGTCAGATCATCTTCG	ATCTCTCAGCTCCACGCCATTG
TGFα	TTGCTGCCACTCAGAAACAGTG	TTGATCTGCCACAGTCCACCTG
iNOS	AGCCTTTGGACCTCAGCAAAGC	TGCCGAGATTTGAGCCTCATGG
TIMP-1	CTTCTGCAATTCCGACCTCGTC	AGGTGGTCTGGTTGACTTCTGG
TIMP-3	CTTCTGCAACTCCGACATCGTG	TGGTGAAGCCTCGGTACATCTTC
GAPDH	AATCCCATCACCATCTTCCA	TGGACTCCACGACGTACTCA

### Immunoblotting and zymography

Protein samples were obtained ether from cell fractions (see above) or total protein was isolated from cell cultures using TriReagent (Sigma-Aldrich, St. Louis, USA). Protein precipitates were dissolved in 1% sodium dodecyl sulfate (SDS) and stored at -80°C. Protein concentrations were determined using the BCA Protein Assay Kit (Thermo Scientific Pierce, Wilmington, USA). Proteins were separated on 10% SDS gels (25 μg per lane) and transferred to a nitrocellulose membrane (Whatman, Maidstone, UK). After blocking with 5% non-fat milk in Tris-Buffered Saline with 0.1% Tween-20 (TBST), immunoblots were probed with the following primary antibodies: anti-ADAM17 (1:1000; R&D, Minneapolis, USA), anti-ERK1/2 and anti-phospho-ERK1/2, anti-c-met, anti-c-Src (all 1:1000; Cell Signaling Technology, Boston, USA), anti-TGFα and anti-TNFα (both 1:1000; R&D, Minneapolis, USA), anti-GAPDH (1:50000; Sigma-Aldrich, St. Louis, USA). Secondary antibodies, rabbit anti-goat and goat anti-rabbit (both 1:10000; Sigma-Aldrich, St. Louis, USA), were peroxidase-conjugated. Signals were detected using ECL plus Western Blotting Detection System (Cell Signaling Technology, Boston, USA) and recorded with a Luminescent Image Analyzer (LAS-3000, Fujifilm Life Science, Düsseldorf, Germany). Densitometry of blots was performed using AIDA Image Analyser Software version 2.2 (Raytest, Straubenhardt, Germany).

The activity of gelatinases was assayed using zymography under nonreducing conditions, as described previously [24,25]. Briefly, conditioned media were precleared by centrifugation (5 min, 12000 × g) and protein concentration was measured by BCA Assay Kit (Thermo Scientific Pierce, Wilmington, USA). Samples were mixed with SDS sample buffer (2.0% SDS, 25% glycerol, 0.1% Bromophenol blue and 60 mM Tris–HCl, pH 6.8) and equal amounts of protein were separated on 8% polyacrylamide gels containing 1 mg/ml gelatine. Gels were washed, incubated for 24 hours in Tris–HCl buffer (50 mmol/l Tris–HCl, 10 mmol/l CaCl_2_, 20 ìmol/l ZnCl_2_, pH 8.0) at 37°C and subsequently stained with Coomassie Blue R-250 (Sigma-Aldrich, St. Louis, USA; 0.125% Coomassie blue R-250, 50% methanol, 10% acetic acid). Regions representing the gelatinase activity of MMP2 and MMP9 were quantified using AIDA Image Analyser Software (Raytest, Straubenhardt, Germany).

### Animal model of BDL-induced acute cholestasis

All animal studies were performed in accordance with European directive 86/609/EEC and were approved by the Czech Central Commission for Animal Welfare. Animals were housed in individually ventilated cages under standard pathogen-free conditions, had ad libitum access to regular chow (Rod18-A10; LASvendi, Soest, Germany) and chlorinated drinking water, and were kept under a 12-hour-dark/12-hour-light cycle. 10-week-old male C57BL/6NCrl mice (Charles Rivers Laboratories, Wilmington, MA) were randomly assigned to four groups: 1) sham followed by administration of vehicle only; 2) sham followed by UDCA administration; 3) BDL followed by administration of vehicle only and 4) BDL followed by UDCA administration. Sham surgery and BDL was performed as described previously [26]. In brief, animals were anesthetized with ketamine (80 mg/kg) and xylazine (10 mg/kg). The common bile duct was exposed through a midline abdominal incision, double-ligated using 4-0 silk, and sectioned between the ligatures. Sham-operated mice had their common bile duct exposed and manipulated but not ligated.

Two days after BDL, mice received either 100 mg/kg UDCA (Sigma-Aldrich, St. Louis, USA) in 0.1 ml of 2.5% sodium bicarbonate (pH 7.4) vehicle daily via orogastric gavage or vehicle alone. These treatments were continued until study completion. Ten days after BDL, mice were anesthetized, blood was obtained by retro-orbital bleed, and animals were sacrificed to evaluate liver damage and related parameters.

Sera were analyzed for alkaline phosphatase (ALP) using a commercial kit (Roche Diagnostics, Prague, Czech Republic). TNFα was assayed with multiplex Bio-Plex mouse array (Bio-Rad Laboratories, Prague, Czech Republic) using the Bio-Plex 200 System (Bio-Rad Laboratories, Prague, Czech Republic). Serum level of mouse sMet was assayed by ELISA using a kit from R&D Systems (R&D Systems, Minneapolis, USA).

### Histology

Liver lobes were fixed in 4% neutral buffered formaldehyde for 24 h at 4°C and processed in an automated tissue processor (Leica Microsystems, Leitz, Germany). To visualize glycogen deposits, tissue sections of 3 μm in thickness were stained with periodic acid of Schiff (PAS) kit (Sigma-Aldrich, St. Louis, USA) according to manufacturer’s instructions. Analysis of the stained slides was performed using a Zeiss Axio Imager microscope (Zeiss Czech Republic, Prague, Czech Republic) equipped with polarization filter and Axiocam ERc5s digital camera. Representative images were captured at 40× magnification and processed consecutively using the GIMP graphic editor version 2.8 (Free Software Foundation, Boston, Massachusetts, USA).

Semiquantitative scoring of PAS staining was made by counting the proportion of PAS-positive and PAS-negative cells. More than five randomly chosen optical fields were evaluated, each with >80 cell per field. Staining was quantitated by blinded scoring on a scale of 0–3+ with: 0, PAS reaction negative; 1+, PAS reaction diffuse and barely detectable or focal of moderate intensity; 2+, PAS reaction diffuse, detectable or strong focal reaction but encompassing less than 50% of the cell cut surface; 3+, PAS reaction diffuse, moderate to strong or strong focal reaction encompassing more than 50% of the cell cut surface.

### Statistical analysis

Statistical analyses were performed with GraphPad Prism software version 5.04 (GraphPad Software, San Diego California, USA). Results from independent experiments were analyzed with two-tailed one-way ANOVA followed by Student-Newman-Keuls post-hoc test. Data are presented as mean values; error bars in figures represent SEM; n values and statistical significance are specified in figure legends.

## Results

### UDCA treatment results in reduction of TNFα, TGFα, and c-Met shedding

To study the effects of UDCA on shedding under conditions reminiscent of the activated state of cells in diseased liver, human hepatoma HepG2 cells were stimulated with phorbol-12-myristate-13-acetate (PMA) that is known to stimulate shedding of TGFα family members [27].

**Figure 1:**
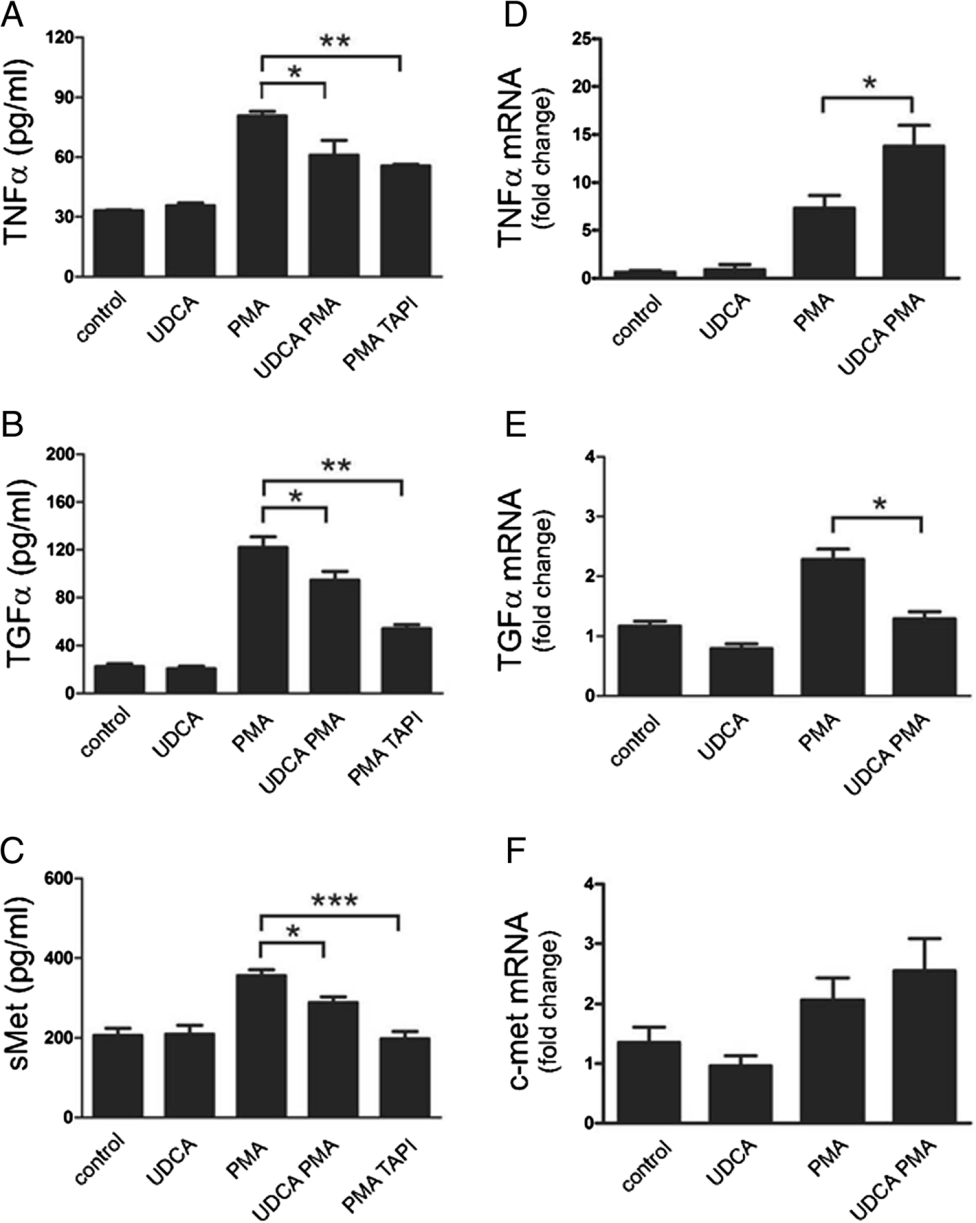
**UDCA reduces shedding of TNFα, TGFα, and c-Met.** HepG2 cells were either left untreated (control), or pretreated with 200 μmol/l UDCA (UDCA), or with 10 nmol/l metalloproteinase inhibitor TAPI-2 (TAPI) for 2 hours. Cells were then either stimulated with 10 nmol/l PMA (PMA) for an additional 24 hours or left non-stimulated. **(A-C)** Levels of human TNFα **(A)**, TGFα **(B)**, and sMet **(C)** in conditioned media were measured by ELISA. **(D-F)** In parallel, the relative expression levels of TNFα **(D)**, TGFα **(E)** and c-Met **(F)** at the mRNA level were followed by qRT-PCR. Expression of genes of interest was normalized to GAPDH and expressed as fold change of control sample (for details see Materials and Methods section). Mean values ± SEM are shown (A-C, n = 3; D-F, n = 5). *p < 0.05; **p < 0.01; ***p < 0.001.

HepG2 cells were pretreated either with UDCA or vehicle only and following 24 hours of activation, conditioned media were analyzed by ELISA for levels of shed TNFα, TGFα, and sMet. Experiments revealed that PMA massively increased shedding of all three substrates (Figure 1A-C) from the cell surface, and this effect was already visible after 2 and 4 hours of PMA stimulation (Additional file 1: Figure S1). However, when the cells were treated with UDCA prior to stimulation, this response was significantly reduced, although UDCA alone had no effect on shedding (Figure 1A-C). While this inhibitory effect was already apparent on released cytokines levels from 2 (TNFα) and 4 (sMet) hours of ongoing PMA stimulation (Additional file 1: Figure S1), only prolonged incubation (24 hours; Figure 1A-C) resulted in significant differences in released levels of all measured substrates. Increased expression of carbonic anhydrase (CA) and inducible nitric oxide synthase (iNOS) in UDCA-treated HepG2 cells indicated UDCA-dependent activation of farnesoid nuclear receptor [28], confirming the proper distribution and mode of action of UDCA (Additional file 2: Figure S2).

To test whether this PMA-mediated shedding can be attributed to proteolytic activity of ADAM17, we used TAPI-2, a specific ADAM17 inhibitor [29]. Pre-treatment of HepG2 cells with TAPI-2 led to significant reduction of release of TNFα, TGFα, and sMet into cell media. Regarding TNFα levels (Figure 1A), the inhibitory effect of TAPI-2 was comparable to that of UDCA (Figure 1A-C) suggesting that UDCA is involved in downregulation of ADAM17 activity, the major TNFα sheddase.

Since PMA has been previously shown to upregulate the expression of many cytokines and proteins [30], we tested its effect on TNFα, TGFα, and c-Met. qRT-PCR analysis of PMA-treated HepG2 cells showed an increase of the mRNA levels of all factors (Figure 1D-F). Surprisingly, pre-treatment of these cells with UDCA resulted in even higher relative expression of TNFα and c-Met (Figure 1D,F). The significant increase in TNFα expression was accompanied by a significant decrease in TNFα release (Figure 1A) suggesting thus that the UDCA treatment indeed employs modulation of shedding activities. Only the TGFα mRNA remained, upon combined treatment with UDCA and PMA, at a level comparable to non-stimulated cells (Figure 1B). Quantitative cell fractionation of non-treated and UDCA-treated HepG2 cells followed by immunoblotting analysis revealed comparable distribution of TNFα, TGFα, and sMet in both samples, thereby excluding possible UDCA interference with the transport of shed substrates to the cell membrane or their internalization (Additional file 3: Figure S3).

**Figure 2:**
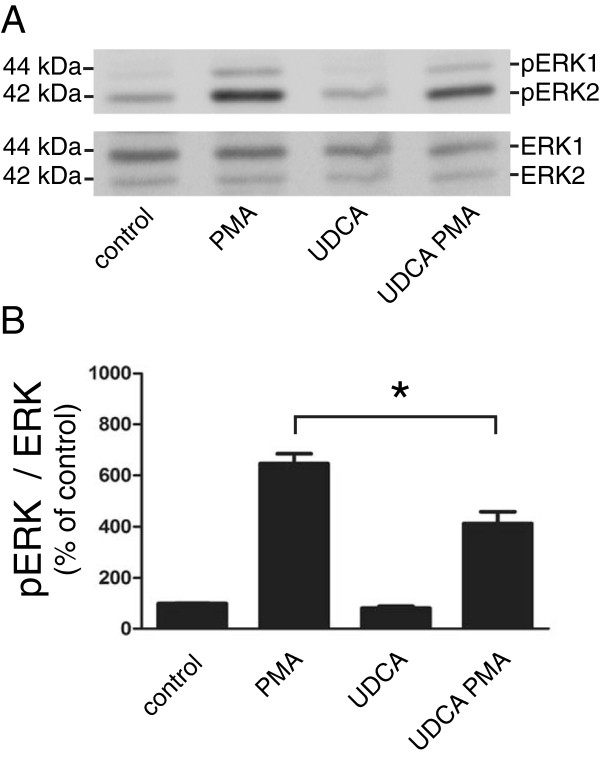
**UDCA treatment decreases ERK1/2 activity.** HepG2 cells were either left untreated (control) or pretreated with 200 μmol/l UDCA (UDCA) or with 10 nmol/l metalloproteinase inhibitor TAPI-2 (TAPI) for 2 hours. Cells were then either stimulated with 10 nmol/l PMA (PMA) for an additional 24 hours or left non-stimulated. **(A)** Cell lysates from treated and control cells were analyzed by immunoblotting with anti-ERK1/2, and anti-phospho-ERK1/2 antibodies. Equal amounts of proteins were loaded in each lane; representative Western blots from three independent experiments are shown. **(B)** Signal intensities of phospho-ERK1/2 bands, which were densitometrically determined in three independent experiments (including the one shown), were normalized to ERK1/2 and compared to values obtained for control cells (100%). Mean values ± SEM are shown (n = 3). *p < 0.05.

To follow the functional impact of UDCA on ADAM17-mediated signaling, we monitored the activation status of mitogen-activated protein kinase 1 and 2 (ERK1/2), the downstream signal of TGFα binding to the EGF receptor [31]. As shown in (Figure 2A,B), immunoblotting analysis using anti-phospho-ERK1/2 antibodies revealed that treatment with UDCA alone had no effect on ERK1/2 activation. However, stimulation of HepG2 cells with PMA resulted in robust increase in ERK1/2 phosphorylation, which was significantly reduced by pre-treatment with UDCA (Figure 2).

### UDCA interferes with ADAM17 maturation

**Figure 3:**
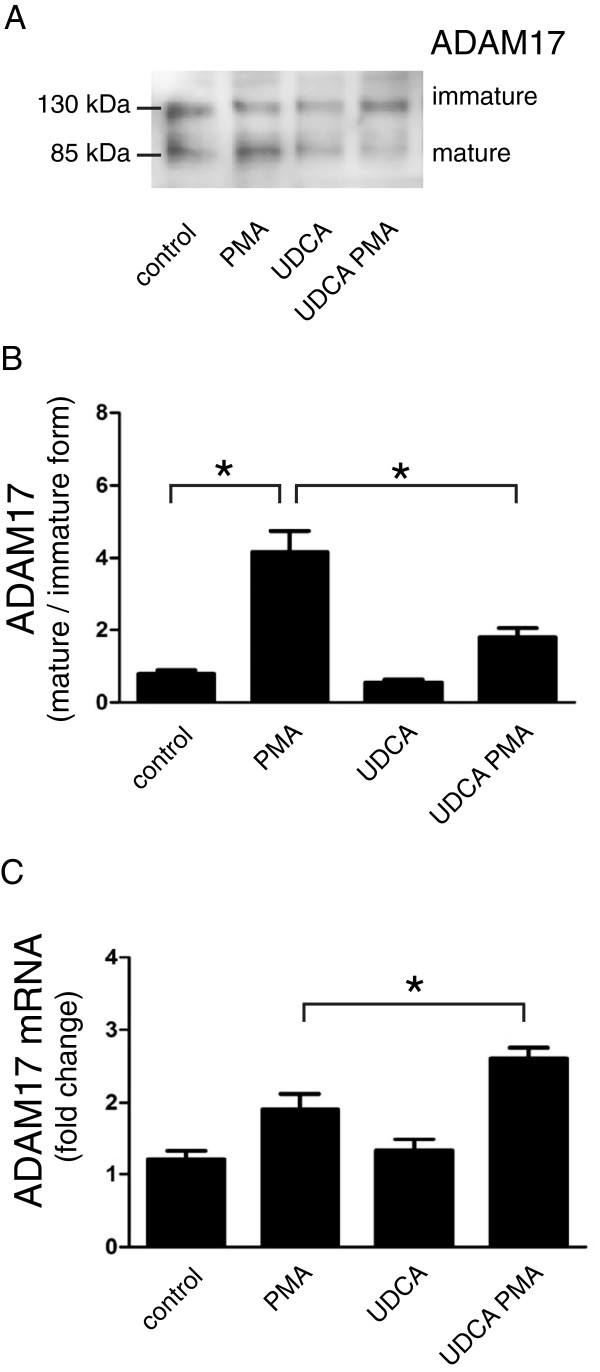
**UDCA interferes with ADAM17 maturation.** HepG2 cells were either left untreated (control) or pretreated with 200 μmol/l UDCA (UDCA) or with 10 nmol/l metalloproteinase inhibitor TAPI-2 (TAPI) for 2 hours. Cells were then either stimulated with 10 nmol/l PMA (PMA) for an additional 24 hours or left non-stimulated. **(A)** Cell lysates from treated and control cells were analyzed by immunoblotting with anti-ADAM17 antibodies. Equal amounts of proteins were loaded in each lane; representative Western blots from three independent experiments are shown. **(B)** Signal intensities of mature ADAM17 bands (85 kDa), which were densitometrically determined in three independent experiments (including the one shown), were normalized to immature ADAM17 form (130 kDa). Mean values ± SEM are shown (n = 3). *p < 0.05; **p < 0.01. **(C)** mRNA was isolated from HepG2 cells as described in the text. The relative expression levels of ADAM17 were followed by qRT-PCR. Expression was normalized to GAPDH and expressed as fold change of control sample. Mean values ± SEM are shown (n = 3). *p < 0.05.

As UDCA significantly decreased the level of soluble TNFα, the substrate of ADAM17 [32], we further addressed the question whether UDCA influences the activation of ADAM17, i.e. whether the formation of the mature form of ADAM17 is affected. The exposure of HepG2 cells to PMA resulted in a pronounced formation of the mature form of ADAM17 whereas the presence of UDCA alone had no effect (Figure 3A,B). However, pre-treatment of cells with UDCA prior to PMA stimulation resulted in a significant decrease in the formation of the mature form of ADAM17 (Figure 3B). Similarly to TNFα expression (Figure 1A), RT-PCR analysis showed that ADAM17 mRNA levels rose after combined PMA and UDCA treatment, but this increase did not lead to a consequent elevation in ADAM17 shedding (Figure 3C).

**Figure 4:**
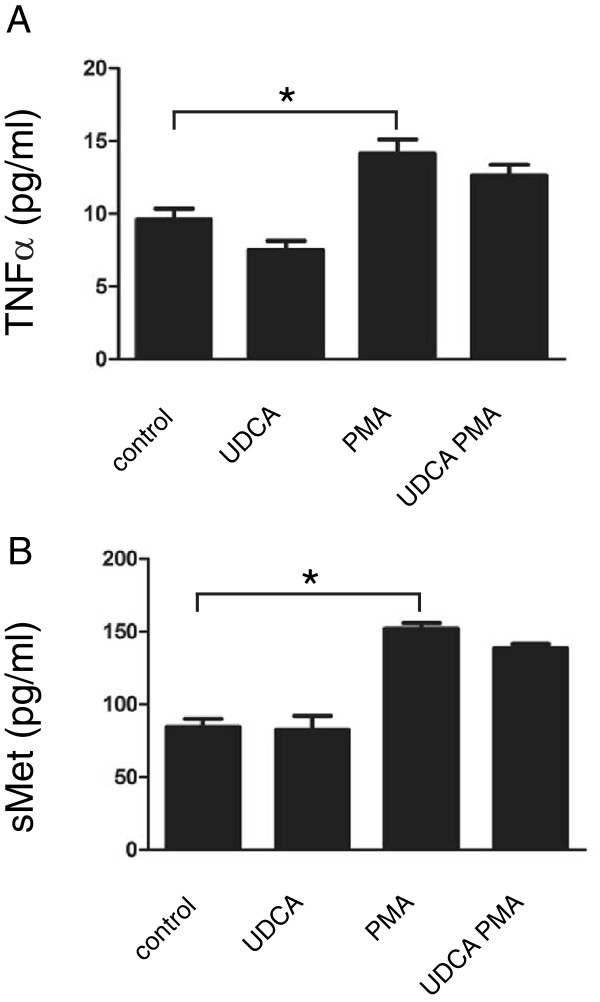
**UDCA reduces TNFα and c-Met shedding in LX2 cells.** LX2 cells were either left untreated (control) or pretreated with 200 μmol/l UDCA (UDCA) or with 10 nmol/l metalloproteinase inhibitor TAPI-2 (TAPI) for 2 hours. Cells were then either stimulated with 10 nmol/l PMA for an additional 24 hours or left non-stimulated. **(A-B)** Levels of human TNFα **(A)** and sMet **(B)** in conditioned media were measured by ELISA. Mean values ± SEM are shown (n = 3). *p < 0.05.

To address the question of how the UDCA inhibitory effect on ADAM17 applies to non-hepatocyte cell types in the liver, human hepatic stellate cells LX2 were pretreated either with UDCA or vehicle only and stimulated for a following 24 hours with PMA. Conditioned media were analyzed by ELISA for levels of shed TNFα and sMet as before. Similar to data obtained with HepG2 cells, these experiments revealed that PMA significantly increased shedding of both substrates (Figure 4A-B) from the cell surface. Treatment with UDCA prior to stimulation resulted in reduction of LX2 cells response (Figure 4A-B) although the effect was not as prominent as for HepG2 cells (compare Figure 1A-C and Figure 4A-B). As UDCA exhibited similar impact on LX2 cells, it is likely that the inhibitory effect of UDCA on ADAM17-mediated release of membrane-bound substrates in PMA-stimulated cells is a general mechanism.

**Figure 5:**
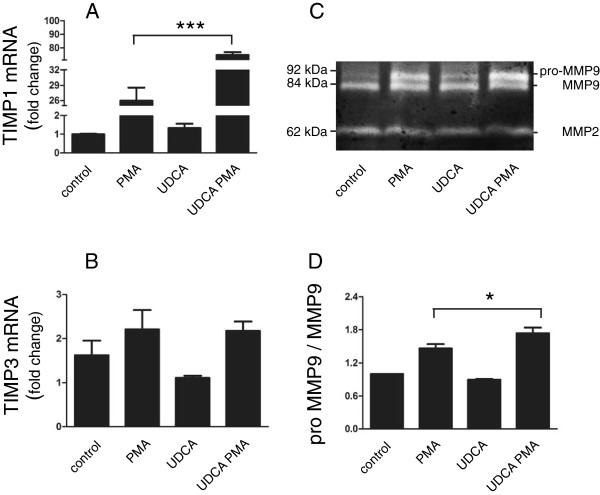
**UDCA-treatment upregulates the expression of TIMP-1 and alters MMP9 activity.** HepG2 cells were either left untreated (control) or pretreated with 200 μmol/l UDCA (UDCA) for 2 hours. Cells were then either stimulated with 10 nmol/l PMA (PMA) for an additional 24 hours or left non-stimulated. **(A,B)** The relative expression levels of TIMP-1 and TIMP-3 were assayed by qRT-PCR. Expression was normalized to GAPDH and expressed as fold change of control sample. Mean values ± SEM are shown (n = 3). ***p < 0.001. **(C)** Conditioned media from treated and control cells were analyzed for the expression and activity of MMP9 and MMP2 using zymography. Equal amounts of proteins were loaded in each lane. **(D)** Band intensities of MMP9 proform (pro-MMP9) were densitometrically determined in three independent experiments (including the one shown) and compared to the active MMP9 form (92 kDa); the diagram shows pro-MMP9/MMP9 ratio. Mean values ± SEM are shown (n = 3). *p < 0.05.

As UDCA treatment in patients with cholestasis and cirrhosis are beneficial to hepatic functions, and this may include also the inhibition of ECM dissolution by MMPs [33-35], we also analyzed whether UDCA influences MMPs. Interestingly, qRT-PCR analysis revealed that mRNA levels of TIMP-1 and -3 increased in cells treated with both PMA and UDCA (Figure 5A,B). However, the UDCA treatment alone had no significant effect on their expression. Since TIMP-1 acts as an endogenous inhibitor of matrix metalloproteinase 9 (MMP9) [36], we next examined its proteolytic activity using zymography. Analysis of conditioned media revealed two clear bands (Figure 5C) corresponding to pro- and active MMP9 forms. The intensities of the 92 kDa band, indicating the pro-form, and the 84 kDa band, indicating the processed/active form, were both clearly elevated upon PMA stimulation when compared to the basal level detected in non-stimulated cells (control). Although the expression of MMP9 appeared even higher after combined treatment with UDCA and PMA, the conversion to active MMP9 84 kDa-form was reduced in comparison to PMA stimulation (Figure 5C,D).

### UDCA administration reduces serum level of ADAM17 substrates and preserves functional activity of hepatocytes in BDL mice

**Figure 6:**
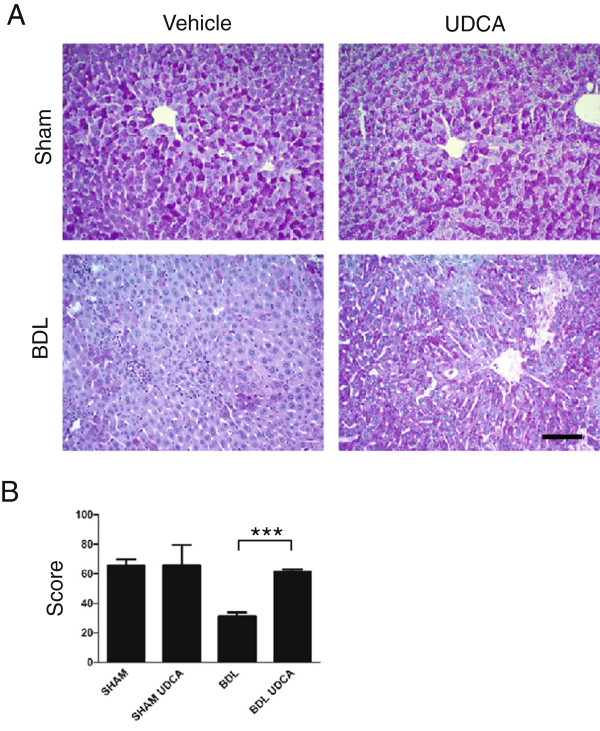
**UDCA treatment results in reduced sMet serum levels and relative liver weight in a mouse model of acute cholestasis. (A)** Schematic representation of a model of BDL-induced acute cholestasis. Acute cholestasis was induced in C57BL/6NCrl mice by common bile duct ligation (for details see Materials and Methods). Serum and the whole liver were collected from sham-operated animals treated with vehicle only (SHAM), sham-operated animals treated with UDCA (SHAM UDCA), animals with ligated bile ducts treated with vehicle only (BDL), and animals with ligated bile ducts treated with UDCA (BDL UDCA). Serum levels of ALP **(B)**, TNFα **(D)** and sMet **(E)** were assessed as described in the text. **(C)** Relative liver weight was calculated as the ratio of liver weight to body weight (100%). Mean values ± SEM are shown (n = 4). *p < 0.05; ***p < 0.001.

To further explore the effects of UDCA on ADAM17-mediated shedding, C57BL/6NCrl mice were subjected to common BDL and subsequently treated with UDCA via orogastric gavage for 6 days (Figure 6A).

**Figure 7:**
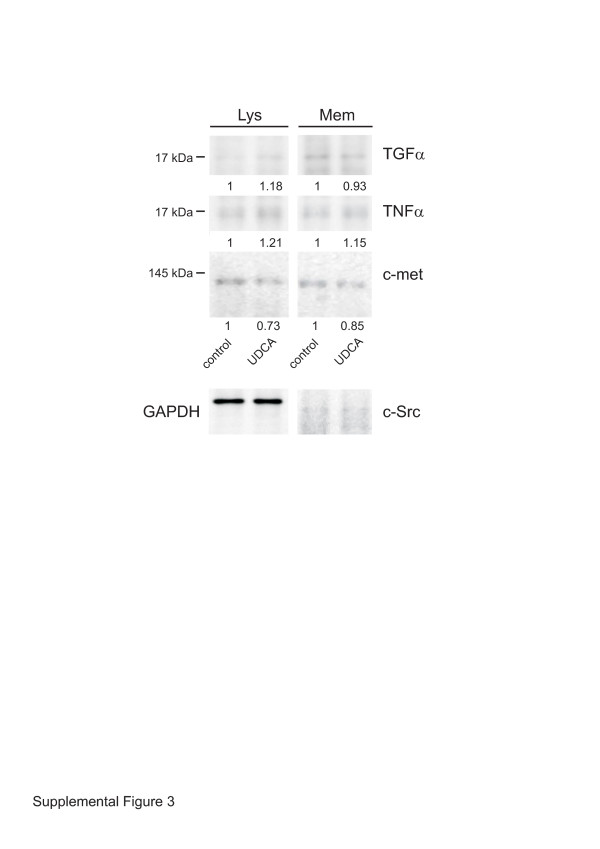
**UDCA treatment preserves PAS-positive collagen aggregates in the liver with acute cholestasis. (A)** Tissue sections of liver isolated from sham-operated animals treated with vehicle only (SHAM), sham-operated animals treated with UDCA (SHAM UDCA), animals with ligated bile ducts treated with vehicle only (BDL), and animals with ligated bile ducts treated with UDCA (BDL UDCA) were stained with PAS for glycogen storage in hepatocytes. Bar, 50 μm. **(B)** Intensity of PAS reaction was semiquantitatively evaluated and normalized as described in the text; >80 cells per field from more than five randomly chosen optical fields of each dissected liver. Mean values ± SEM are shown (n = 4). ***p < 0.001.

The body weights of 8 day BDL and SHAM animals treated with UDCA or vehicle were significantly lower than the control group of untreated animals. However, consistent with previously published results, UDCA had a beneficial effect on the course of acute cholestasis [37-39]. UDCA administration attenuated hepatocellular damage as documented by significantly reduced ALP levels in serum (Figure 6B), decreased relative liver weight, the reliable indicator of hepatic injury (Figure 6C), and by histochemical analysis (Figure 7A,B).

Under these conditions we evaluated serum levels of two known ADAM17 substrates, TNFα and sMET, which are linked to development of liver injury (Figure 6D,E). ELISA analyses showed that the level of sMet was significantly reduced in BDL animals treated with UDCA compared to untreated BDL group (Figure 6E). Similar effects, however less pronounced, were observed upon administration of UDCA in sham-operated groups (Figure 6E). Such reduction of sMet levels is not only fully consistent with moderate liver injury, but also likely reflects the lower activity of ADAM17 in livers of UDCA-treated animals. Although not significant, a similar effect or tendency was also seen in TNFα levels in BDL animals treated with UDCA (Figure 6D). Generally, TNFα levels were higher in all experimental groups, including the sham controls, compared to control animals (Additional file 4: Figure S4). This can probably be attributed to acute inflammation after surgery.

Histological analysis of liver sections further supported these findings. Period acid Schiff (PAS) staining revealed that the hepatocytes of UDCA-treated BDL animals retained substantially greater amounts of intracellular glycogen than did those of the untreated BDL group (Figure 7A,B). In fact, the staining intensity of preserved intracellular glycogen granules after UDCA administration was indistinguishable from those observed in sham-operated animals (Figure 7A,B), suggesting comparable metabolic activity of the hepatocytes.

Taken together, these findings indicate that the inhibition of ADAM17 in response to UDCA treatment can provide an additional mechanistic explanation for the hepatoprotective effects of UDCA in acute cholestasis.

## Discussion

UDCA is currently only approved by the FDA to treat primary biliary cirrhosis (PBC), however, it exhibits no benefit in patients with primary sclerosing cholangitis (PSC) [40,41]. UDCA-treatment of PBC patients results in a decrease of serum markers of hepatic damage and its beneficial effect is believed to be based on its cytoprotective, anti-apoptotic, anti-oxidative, and immunomodulating functions [22]. However, the mechanism of the UDCA impact is still fragmentary.

Although regulation of TNFα levels after UDCA treatment has been documented in patients as well as in rodent models [21,22], there are no reports about the mechanism how UDCA influences its bioavailability. Unlike other proinflammatory factors such as IL-1 and IL-6, TNFα must be released from the cell-surface via a process termed ectodomain shedding [42,43]. This shedding controls also bioavailability of factors belonging to the TGFα family and, thus, this process is of pivotal importance for liver pathophysiology as many signaling mediators such as TNFα, TGFα, and others [21,22] need to be released from the cell membrane to be active as ligands [15,27,42].

In this work we focused on TNFα, TGFα, and sMet, the factors that are released from the cell-surface due to the shedding activity of the ADAM family of metalloproteinases. The data shown here clearly show that UDCA decreases the levels of ADAM17 substrates and that this reduction is due to an inhibition of ADAM17 maturation.

Inflammation inducers such as bacterial lipopolysacharides, ceramide, or PMA induce shedding by activation of metalloproteinase 7 (MMP7) and ADAM17 [44,45]. Although the exact biological significance of ligand or receptor shedding is unclear in most liver pathologies, it is widely accepted that the level of TNFα is hallmark of disease progression and that UDCA treatment is beneficial for liver regeneration and the reduction of inflammation [22]. This is also supported by our findings that UDCA treatment reduced TNFα shedding. In spite of the combination of UDCA and PMA increasing the mRNA level of TNFα more than PMA alone, the amount of TNFα in cell medium did not increase. Based on these findings, experiments with the ADAM17 specific inhibitor TAPI-2 supported the conclusion that UDCA blocks activity of ADAM17 by inhibiting the formation of the mature form of ADAM17.

TGFα, which is produced in hepatocytes and released by ADAM17 during liver regeneration, is part of an early cytokine and growth factor response and one of the essential ligands for EGFR stimulation. The activation of EGFR promotes cell proliferation and survival, via signaling through the ERK pathway [42]. We found that levels of TGFα are reduced in cells pre-incubated with UDCA and stimulated with PMA. As a functional consequence of the lower activity of ADAM17, TGFα release and EGFR activation were reduced as demonstrated by lower phosphorylation of ERK.

Hepatocyte growth factor and its receptor c-Met represent the main proliferative axis in hepatocytes. It has been proposed that c-Met receptor is shed by ADAM10 [46] and recently it was also reported to be an ADAM17 substrate [47]. Shedding of c-Met differs from EGFR or cytokines release, as it causes inactivation of receptor and eliminates its signaling [48]. In comparison to TNFα and TGFα, sMet exhibited the most sensitive change to UDCA treatment. Also the ectopic expression of ADAM17 without PMA stimulation already increased sMet concentration in media. Inhibition of ADAM17 and releasing of sMet by TAPI-2 confirmed previously reported c-Met shedding by ADAM17 in LX-2 cells [24]. The elevated sensitivity of c-Met towards PMA and UDCA treatment could be caused by higher expression of c-Met in hepatocytes, about ten times more than TNFα and TGFα.

The UDCA-dependent decrease of TNFα serum levels due to the inhibition of ADAM17 activity, likely has a beneficial effect as TNFα exhibits strong pro-inflammatory effects. However, inhibition of the release of TGFα and sMet might have some adverse effect on liver function as these factors together with their receptors are crucial for liver regeneration (for review see [49]). Thus, our findings based on UDCA-mediated inhibition of the shedding activity of ADAM17 may explain why, for instance, UDCA exhibits low or no beneficial effect on primary sclerosing cholangitis and other chronic liver diseases [1,50]: inhibiting the activity of ADAM17 appears to result in diminished inflammatory reactions but, in parallel, it may exhibit adverse effects due to inhibition of c-Met and EGFR signaling on liver regeneration and function.

## Conclusions

In the present study we demonstrate that UDCA affects the activity of ADAM17, which in turn results in decreased shedding of ADAM cell-surface bound factors such as TNFα, TGFα, and c-Met. UDCA treatment also increases the expression of matrix metalloproteinase inhibitor TIMP-1, thereby preventing MMPs from their deteriorative proteolytic activity in the liver. Altogether, these results identify ADAM17 as a novel target of UDCA in hepatocytes and study improve our overall understanding of UDCA treatment and its beneficial effects.

## Competing interests

The authors declare that they have no competing interests.

## Authors’ contributions

KC, MJ, MG and designed and executed the studies. HB, KC, OZ and IK carried out the experiments in vitro. MG and OZ carried out the animal experiments. KC and MG drafted the manuscript and prepared figures. RS contributed to study concept, study design, and writing the manuscript. All authors read and approved the final manuscript.

## Pre-publication history

The pre-publication history for this paper can be accessed here:

http://www.biomedcentral.com/1471-230X/13/155/prepub

## Supplementary Material

Additional file 1: Figure S1 UDCA reduces shedding of TNFα and c-Met in PMA-stimulated cells. HepG2 cells were either left untreated (control), or pretreated with 200 μmol/l UDCA (UDCA) for 2 hours. Cells were then either stimulated with 10 nmol/l PMA (PMA) for either 2 (A,B) or 4 (C,D) hours, or left non-stimulated. Levels of human TNFα (A,C) and sMet (B,D) in conditioned media were measured by ELISA. Mean values ± SEM are shown (n = 3). *p < 0.05. Click here for file

Additional file 2: Figure S2 UDCA-treated HepG2 cells exhibit increased expression of carbonic anhydrase (CA) and inducible nitric oxide synthase (iNOS). HepG2 cells were either left untreated (control) or pretreated with 200 μmol/l UDCA (UDCA) for 2 hours. Cells were then either stimulated with 10 nmol/l PMA (PMA) for an additional 24 hours or left non-stimulated. Relative expression levels of carbonic anhydrase (CA; A) and inducible nitric oxide synthase (iNOS; B) were assayed by qRT-PCR. Expression of both genes was normalized to GAPDH and expressed as fold change of control sample (for details see Materials and Methods section). Mean values ± SEM are shown (n = 3). **p < 0.01. Click here for file

Additional file 3: Figure S3 UDCA treatment does not affect distribution of TNFα, TGFα, and c-Met between membrane and cytoplasmic compartments. Tolal lysate (Lysate) and membrane subfractions of non-treated (control) and UDCA-treated (UDCA) HepG2 cells normalized for equal protein contents, were immunoblotted using antibodies to TNFα, TGFα, and c-Met. GAPDH and c-Src were used as loading controls. Click here for file

Additional file 4: Figure S4 Sham animals have elevated inflammation markers. Acute cholestasis was induced in C57BL/6NCrl mice by common bile duct ligation. Serum and the whole liver were collected from sham-operated and from animals without any surgery. Serum levels of ALP (A), sMet (C) and TNFα (D) were assessed as described in materials and methods. (B) Relative liver weight was calculated as the ratio of liver weight to body weight (100%). Mean values ± SEM are shown (n = 4). **p < 0.01. Click here for file
