# Supplementary figures and images for: Liver protective effect of ursodeoxycholic acid includes regulation of ADAM17 activity
Source: BMC Gastroenterol. 2013 Oct 30;13:155. doi: 10.1186/1471-230X-13-155 (PMC3835136; doi:10.1186/1471-230X-13-155)

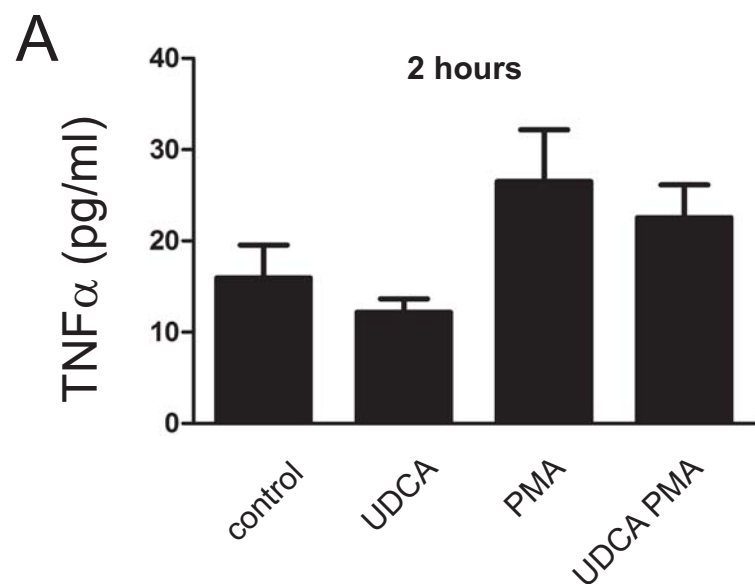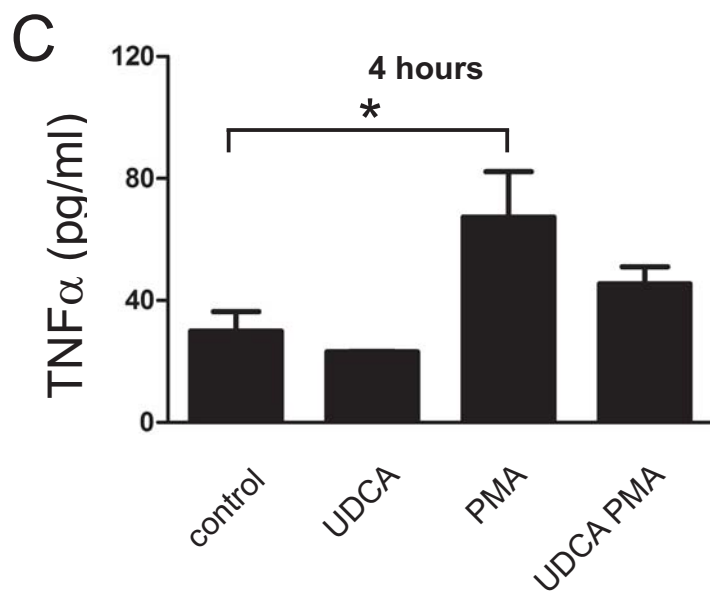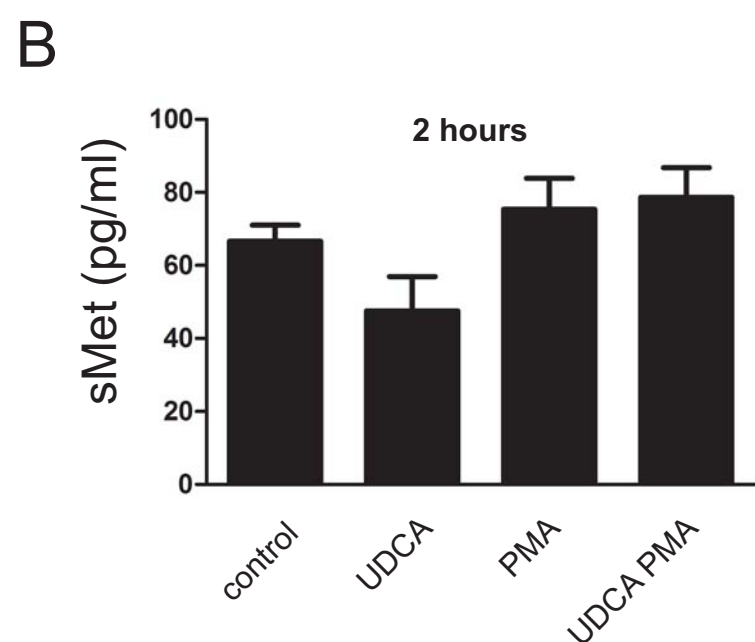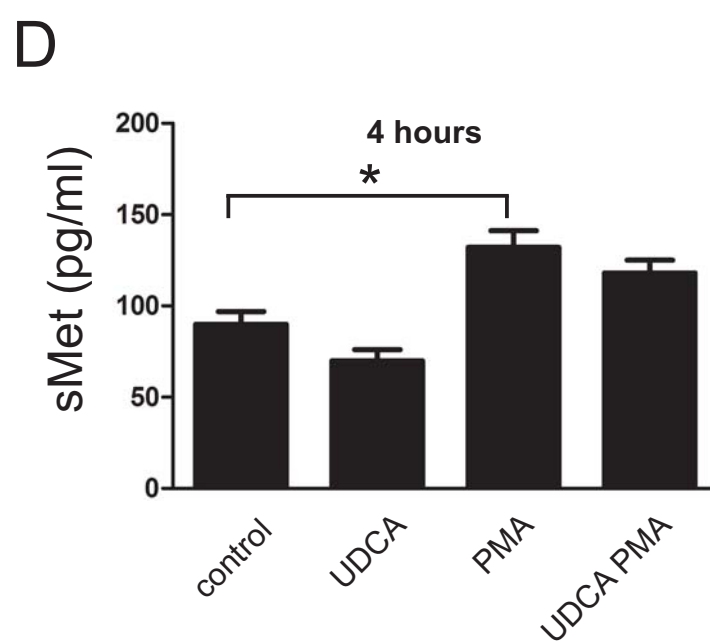

Supplement: Additional file 1: Figure S1 — UDCA reduces shedding of TNFα and c-Met in PMA-stimulated cells. HepG2 cells were either left untreated (control), or pretreated with 200 μmol/l UDCA (UDCA) for 2 hours. Cells were then either stimulated with 10 nmol/l PMA (PMA) for either 2 (A,B) or 4 (C,D) hours, or left non-stimulated. Levels of human TNFα (A,C) and sMet (B,D) in conditioned media were measured by ELISA. Mean values ± SEM are shown (n = 3). *p < 0.05. [file 1471-230X-13-155-S1.pdf]

A

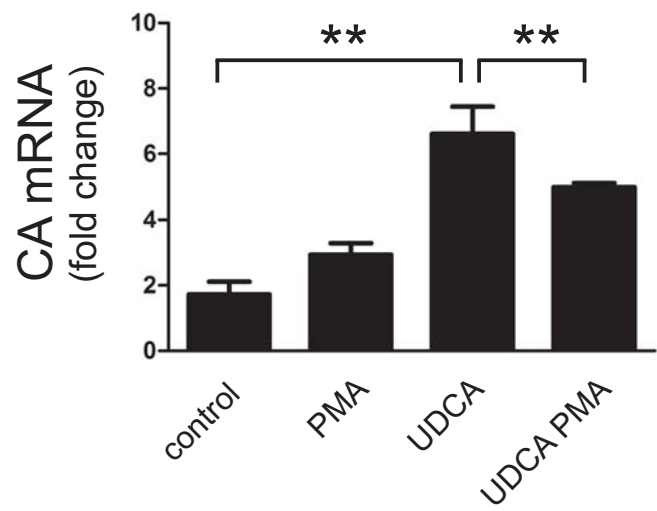

B

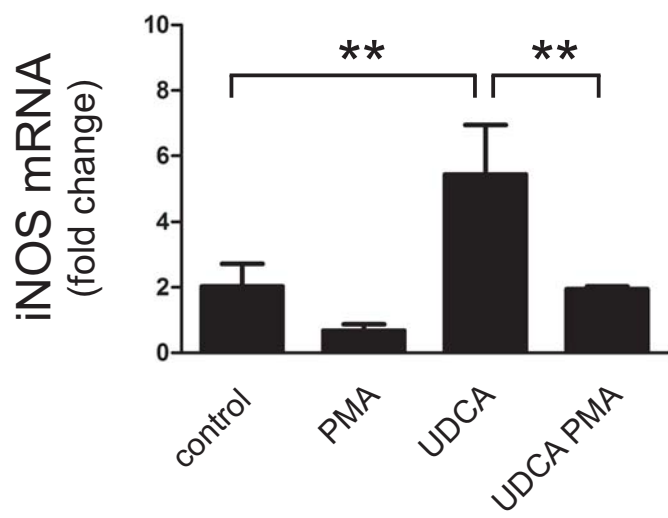

Supplemental Figure 2

Supplement: Additional file 2: Figure S2 — UDCA-treated HepG2 cells exhibit increased expression of carbonic anhydrase (CA) and inducible nitric oxide synthase (iNOS). HepG2 cells were either left untreated (control) or pretreated with 200 μmol/l UDCA (UDCA) for 2 hours. Cells were then either stimulated with 10 nmol/l PMA (PMA) for an additional 24 hours or left non-stimulated. Relative expression levels of carbonic anhydrase (CA; A) and inducible nitric oxide synthase (iNOS; B) were assayed by qRT-PCR. Expression of both genes was normalized to GAPDH and expressed as fold change of control sample (for details see Materials and Methods section). Mean values ± SEM are shown (n = 3). **p < 0.01. [file 1471-230X-13-155-S2.pdf]

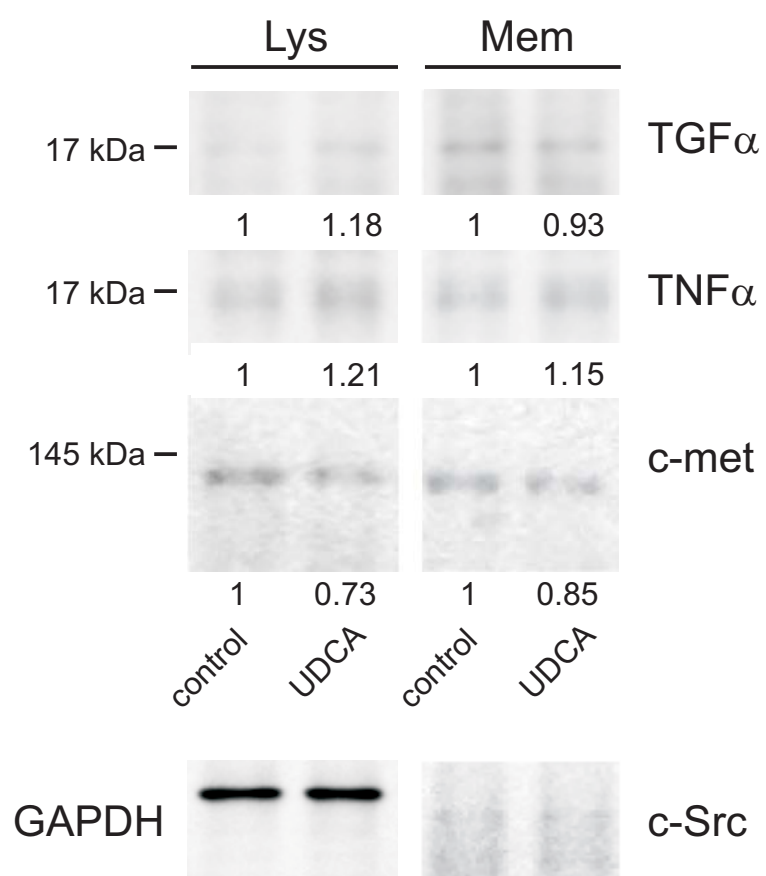

Supplemental Figure 3

Supplement: Additional file 3: Figure S3 — UDCA treatment does not affect distribution of TNFα, TGFα, and c-Met between membrane and cytoplasmic compartments. Tolal lysate (Lysate) and membrane subfractions of non-treated (control) and UDCA-treated (UDCA) HepG2 cells normalized for equal protein contents, were immunoblotted using antibodies to TNFα, TGFα, and c-Met. GAPDH and c-Src were used as loading controls. [file 1471-230X-13-155-S3.pdf]

A

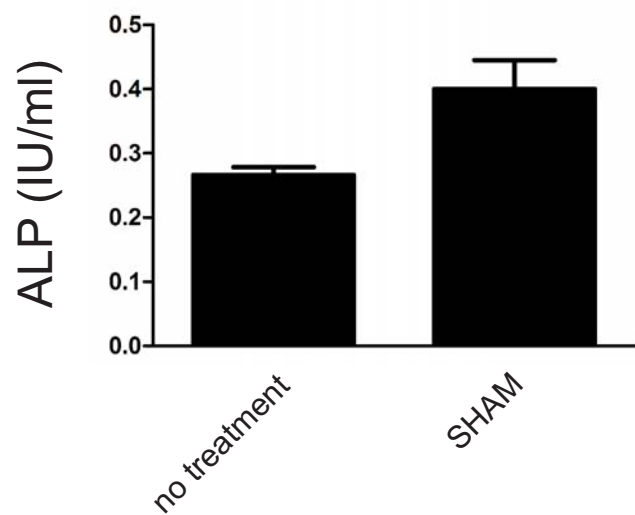

B

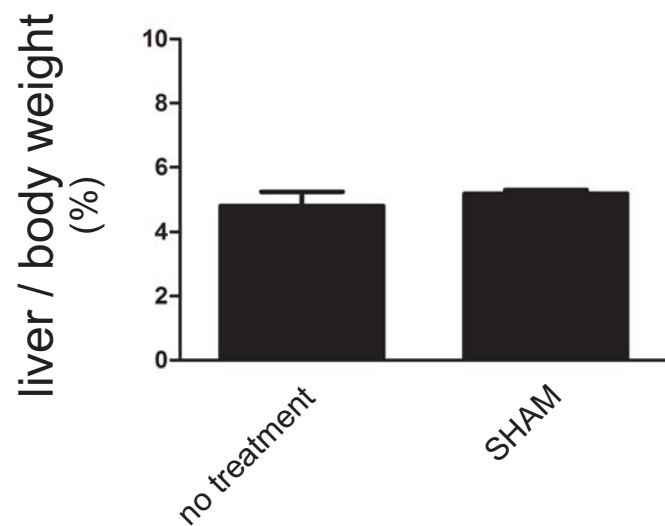

C

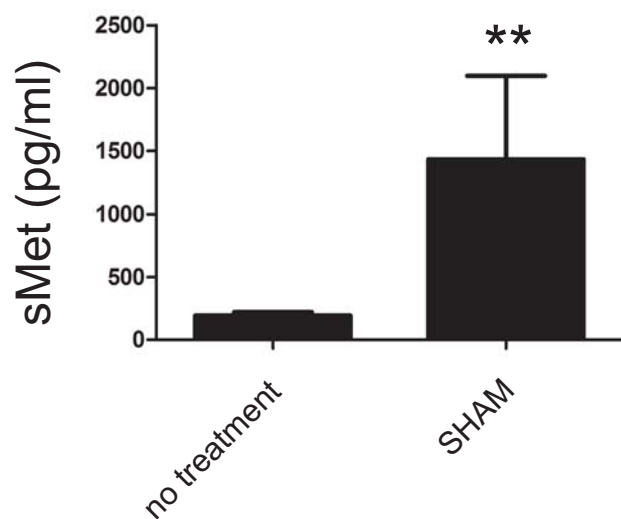

D

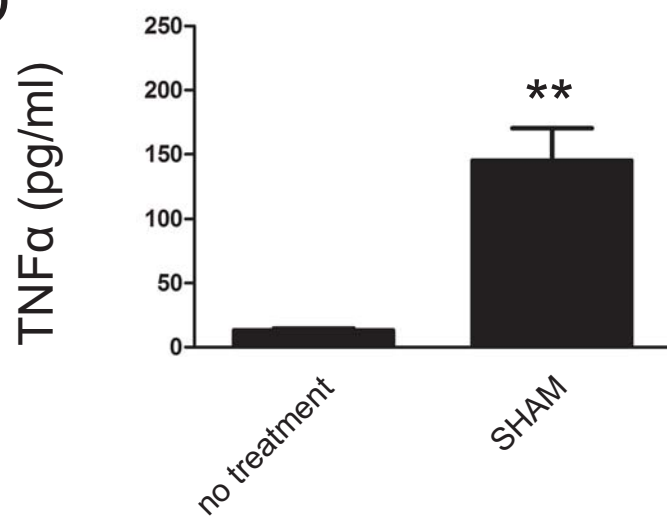

Supplement: Additional file 4: Figure S4 — Sham animals have elevated inflammation markers. Acute cholestasis was induced in C57BL/6NCrl mice by common bile duct ligation. Serum and the whole liver were collected from sham-operated and from animals without any surgery. Serum levels of ALP (A), sMet (C) and TNFα (D) were assessed as described in materials and methods. (B) Relative liver weight was calculated as the ratio of liver weight to body weight (100%). Mean values ± SEM are shown (n = 4). **p < 0.01. [file 1471-230X-13-155-S4.pdf]
